# The giant tear of retinal pigment epithelium following focal laser in central serous chorioretinopathy

**Published:** 2020

**Authors:** Avadhesh Oli, Divya Balakrishnan

**Affiliations:** * Smt. Kanuri Santhamma Centre for Vitreoretinal Diseases, LV Prasad Eye Institute, Banjara Hills Hyderabad India

**Keywords:** RPE tear, RPE rip, CSCR, focal laser in CSCR

## Abstract

A 35-year-old male smoker presented with reduced vision in OD for the previous two years. He reported a further drop in the vision for the previous two weeks after he underwent focal laser elsewhere. Clinical examination and multimodal imaging confirmed a giant tear of the retinal pigment epithelium (RPE) and a focal leak of CSCR. He was managed with the focal laser to the active leak and lifestyle modification. The ocular condition remained stable.

RPE rips in CSCR have been reported to occur spontaneously or with an intervention like exogenous use of steroids, or photodynamic therapy (PDT). This case highlighted the fact that focal laser can precipitate RPE rip in a case of CSCR with tense bullous PED. The physician should keep this fact in mind while counselling the patient before a laser procedure and suspect an RPE rip in a patient with CSCR who presents with an acute onset of vision loss.

## Introduction

RPE tears or rips are commonly associated with choroidal neovascular membrane (CNVM) and pigment epithelial detachment (PED) in patients with age-related macular degeneration [**1**]. The incidence and reporting of RPE rip has increased further with the extensive use of anti-VEGF injections [**2**]. RPE rips associated with central serous chorioretinopathy (CSCR) are uncommon, yet bullous variant of CSCR can present with RPE rip [**3**]. RPE tears associated with CNVM are caused by contraction of CNVM at the edge of PED; on the contrary, increased hydrostatic pressure in the PED is implicated for the blowout of RPE in CSCR [**1**]. The incidence of CNVM related RPE tear is reported to be 20% [**2**]. In an isolated case report, PDT has been associated with RPE tear in a case of CSCR [**4**]. Moreover, the continued use of steroids can worsen the PED in CSCR, leading to RPE tear [**5**]. 

Few isolated case reports have described RPE rips following epidural steroids and photodynamic therapy (PDT); however, we did not come across any report on RPE tear following thermal laser in CSCR.

## Case presentation

Case: A 35-year-old male presented with a decrease in vision in both eyes with intermittent metamorphopsia of 2 years duration. He did not complain of pain, redness, photophobia, flashes or floaters. He was diagnosed with CSCR in both eyes and had recently received focal laser in OD elsewhere. He noticed a sudden reduction in vision in OD after the focal laser. Presenting visual acuity was 20/ 200 and 20/ 20. The anterior segment and intraocular pressure in both the eyes were normal.

**Fig. 1A:**
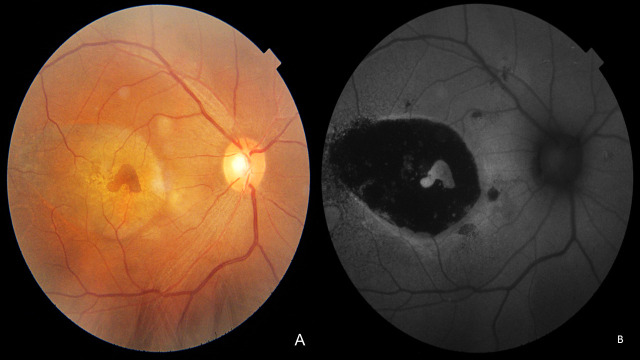
OD fundus showing a 3 DD slate grey area with central pigmentation with RPE changes **Fig. 1B** FAF shows hypo autofluorescence corresponding to an area devoid of RPE and hyper autofluorescence in the centre (curled RPE)

**Fig. 2A:**
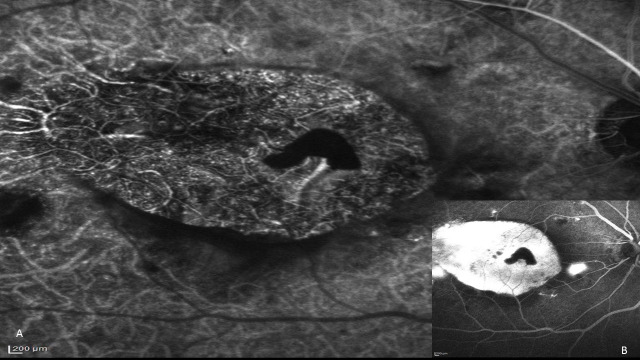
The bare choroid with hyper fluorescence from choriocapillaris is clearly visible in the ICG **Fig. 2B** Window defect with a focal leak on fundus fluorescein angiography

**Fig. 3:**
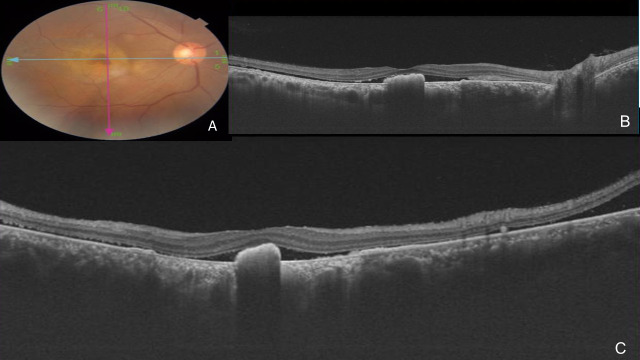
Optical coherence tomography (OCT) passing through fovea (**Fig. 3A**) confirmed the tear in RPE with bunching of RPE with backshadowing (**Fig. 3B, C**) and thickened choroid

The fundus photograph showed a slate grey area of 3 DD with central pigmentation in OD and RPE changes in both the eyes (**Fig. 1A**). Autofluorescence (AF) imaging confirmed the giant RPE tear in OD with hypo AF at the area of the tear with minimal hyper AF at the centre and the edge of the tear due to the rolled-out edges of RPE (**Fig. 1B**). Fundus fluorescein angiography (FFA) showed hyper fluorescence due to window defect at the RPE tear area with a focal leak (**Fig. 2B**), and indocyanine green angiography (ICG) showed hyper fluorescence at the area of RPE tear due to bare choroid (**Fig. 2A**). Optical coherence tomography (OCT) passing through the fovea (**Fig. 3A**) confirmed the tear in RPE with bunching of RPE with backshadowing (**Fig. 3B, C**) and thickened choroid.

RPE tear in OD was diagnosed on multimodal imaging. FFA showed focal leak in OD, and focal laser was performed to the active leak. He was advised to quit smoking and adopt a lifestyle modification and do yoga exercises.

The vision remained stable, and the subretinal fluid resolved at one year follow up.

## Discussion

This case highlighted that in CSCR, the focal laser could induce an RPE rip leading to a sudden reduction in visual acuity.

The vision loss depends on the location and extent of the RPE tear. The RPE tear involving the fovea can cause substantial vision loss. The healing takes by either scarring or migration of RPE cells [**6**]. The prognosis of CSCR related RPE tear is considered better than CNVM related RPE tear [**7**]. 

No definitive treatment guidelines exist for the management of RPE tear. In our patient, we found an active focal leak in FFA and so decided to treat with focal laser because the focal leak would have further compromised RPE with fluid overload. Specific predictive markers have been described for RPE rip formation like the PED height, change in reflectance based on autofluorescence, subretinal cleft formation and duration of PED [**8**]. It is crucial to identify those cases that have the risk of RPE tear like a bullous variant of CSCR [**3**]. Patients should be counselled regarding the risk of RPE tear following laser, especially in high-risk cases. Utmost care should be taken for laser delivery, as the RPE is tense and high energy with small spot size can tear the RPE.

## Conclusion

This report highlighted a rare case of RPE tear in CSCR induced by focal laser. A high index of suspicion of RPE tear should be kept in mind in a case of CSCR if the patient presents with a sudden reduction of vision after the laser procedure. Multimodal imaging helps in early diagnosis and regular follow-up of the patient. Proper patient counselling before focal laser, especially in high-risk cases with a tense PED, must be done.

**Conflict of interest**

The authors declare no conflict of interest. 

**Financial Disclosure**

Nil financial disclosures.

No public private support.
